# Effects of Capsicum and Propyl-Propane Thiosulfonate on Rumen Fermentation, Digestion, and Milk Production and Composition in Dairy Cows

**DOI:** 10.3390/ani10050859

**Published:** 2020-05-15

**Authors:** Andreas Foskolos, Alfred Ferret, Adriana Siurana, Lorena Castillejos, Sergio Calsamiglia

**Affiliations:** 1 Animal Nutrition and Welfare Service (SNiBA), Departament de Ciència Animal i dels Aliments, Universitat Autònoma de Barcelona, 08193 Bellaterra, Spain; andreasfosk@hotmail.com (A.F.); Alfred.ferret@uab.cat (A.F.); adrisiuri@gmail.com (A.S.); lorena.castillejos@uab.cat (L.C.); 2 Department of Animal Science, University of Thessaly, Campus Gaiopolis, 411 10 Larisa, Greece

**Keywords:** capsicum oleoresin, propyl-propane thiosulfonate, digestibility, milk production

## Abstract

**Simple Summary:**

Essential oils and their active compounds have been investigated as feed additives to improve performance in a wide range of farm animals. Most studies related to ruminants have been conducted in vitro and suggest that their effects may be beneficial for ruminant health and production performance. However, in vivo studies are limited. We investigated the effects of capsicum oleoresin (CAP) and propyl-propane thiosulfonate (PTSO) on rumen fermentation, total tract digestibility, and milk yield and composition in lactating dairy cattle. At the doses used, CAP and PTSO failed to demonstrate any effects on rumen fermentation or milk yield, but dry matter intake tended to increase in cows fed with CAP. Feeding PTSO increased milk efficiency compared with the control diet.

**Abstract:**

Essential oils may affect rumen fermentation, nutrient digestion, and milk production and composition. The objective of this study was to test the effects of capsicum oleoresin (CAP) and propyl-propane thiosulfonate (PTSO) on rumen fermentation, total tract digestibility, and milk yield and composition in lactating dairy cattle. Six lactating Holstein cows (averaging (mean ± SD) 130 ± 40 days in milk and 723 ± 55 kg of body weight) fitted with rumen cannulae were used in a duplicated 3 × 3 Latin square design. Treatments were: a control diet (CTR), the CTR diet with the addition of 500 mg/d/cow of CAP, and the CTR diet with the addition of 250 mg/d/cow of PTSO. Dry matter intake (DMI) averaged 20.7 kg/d with a tendency towards higher intake in cows fed CAP and lower in those fed PTSO (*p* = 0.08). Milk yield averaged 31.8 kg/d with no difference among treatments. However, feed efficiency was higher in PTSO supplemented cows compared with CTR (1.65 and 1.41 kg of milk yield/kg of DMI, respectively; *p* < 0.01). At the doses used in this experiment, CAP and PTSO failed to demonstrate any effects on rumen fermentation, but PTSO increased the efficiency of feed utilization to produce milk.

## 1. Introduction

The ban on use of antibiotics as growth promoters in animal feeds in the European Union [1] and the necessity to improve nutrient efficiency at the farm level to reduce the environmental impact of dairy farming [2] have stimulated research on potential alternatives. Essential oils (EO) seem promising as modifiers of rumen fermentation because of their antimicrobial properties [3,4]. Capsicum oleoresin (CAP) has been reported to affect rumen microbial fermentation in vitro with high concentrate diets and low pH [5]. However, changes in rumen fermentation measured in heifers fed high concentrate diets have been small and contradictory [6,7,8]. In contrast, the effect on dry matter (DM) intake has been consistent and increased around 9% to 14% at doses from 125 to 1000 g/d [6,7,8]. Tager and Krause [9] reported that 250 mg capsicum oleoresin had no effect on dry matter intake (DMI) and milk yield. Oh et al. [10] reported no effect of increasing doses of capsicum oleoresin (0, 250, 500, and 1000 mg/day) on DMI in dairy cattle but demonstrated a quadratic response in milk yield. More recently, Oh et al. [11] reported that capsicum tended to increase milk production when feeding 200 mg/d of a rumen protected capsicum oleoresin to dairy cows, as a result of a post-ruminal effect mediated through insulin.

Garlic oil is a complex mix of many different compounds present in the plant or derived from processing and has antimicrobial activities against a wide spectrum of bacteria [4]. Several in vitro batch culture and dual flow continuous culture fermenter trials reported that garlic oil and its main compounds reduced the concentrations of acetate, branch-chained volatile fatty acids, and ammonia-nitrogen, and increased concentrations of propionate and butyrate [12,13]. However, garlic active components are oxygen sensitive and, consequently their effectiveness is reduced in field conditions [14,15]. Propyl-propane thiosulfonate (PTSO) is a modified oxygen-stable organosulfurate compound derived from garlic that can be used in normal production processes without losing its activity. In a previous study, we reported its potential to modify ruminal fermentation in a direction consistent with higher propionate molar proportion, higher outflow of unsaturated fatty acids (UFA), and lower trans-10 cis-12 CLA in an effective dose between 50 and 100 mg/L using a dual flow continuous culture system [16]. In a follow up study with the same in vitro system, 90 mg/L of PTSO reduced the concentration of C18:0, suggesting an inhibition of biohydrogenation [17].

We hypothesized that the addition of CAP might increase DM intake and PTSO may modify rumen fermentation, thus increasing dairy cattle performance. The objective of the current study was to investigate the effects of capsicum oleoresin and propyl-propane thiosulfonate on the rumen fermentation profile, nutrient digestion, and milk yield and composition in dairy cows.

## 2. Materials and Methods

The experiment was conducted at the experimental farm of the Universitat Autònoma de Barcelona and all experimental procedures were approved by the Animal Care and Use Committee (Directive 2010/63/EU; Code CEEAH-00671).

### 2.1. Animals and Experimental Design

Six multiparous Holstein dairy cows fitted with rumen cannulae (10 cm internal diameter, Reference #2C; Bar Diamond Inc., Parma, ID, USA) and averaging (mean ± SD) 130 ± 40 days in milk and 723 ± 55 kg of body weight were enrolled in a duplicated 3 × 3 Latin square design. Each experimental period lasted 31 d, the first 21 d for adaptation to treatments and the last 10 d for sampling. At the beginning of the trial, cows within each square were assigned randomly to one of three treatments: a control diet (CTR); the CTR diet with the addition of 500 mg/d/cow of capsicum oleoresin (CAP); and the CTR diet with the addition of 250 mg/d/cow of propyl-propane thiosulfonate (PTSO). The CAP and PTSO were supplied encapsulated by iso-fusion technology (Pancosma SA, Geneva, Switzerland). The dose of CAP was based on previous studies in heifers [7,8], and the dose of PTSO was based on data extrapolated from in vitro trials using PTSO [16] and in vivo trials using garlic powder [18]. The compounds were mixed with the concentrate daily before they were mixed with forages to form the total mixed ratio (TMR). Cows were housed in tie stalls, fed ad libitum individually once daily after the morning milking with amounts to ensure 5 to 10% refusals, and had continuous access to water. The TMR diet was formulated with the Cornell Net Carbohydrate and Protein System (CNCPS. V6.5, Cornell University) [19] to meet requirements for 30 kg of milk/d at predicted DMI of 20 kg/d (Table 1).

### 2.2. Sampling Procedures

#### 2.2.1. Milk Production and Composition

Cows were machine-milked twice daily (7:00 and 16:00) and milk production was recorded for three consecutive days at the beginning of the sampling period. Milk samples were collected from each cow on days 22, 23, and 24 of each experimental period, preserved with potassium dichromate (0.3 g/L) and stored at 4 °C until analyses. Milk composition was analyzed in each sample and expressed on a daily basis proportionally to the actual milk weight of each milking. Another sample was frozen at −20 °C without preservative for milk FA profile. Milk production was expressed as 3.5% fat corrected milk (FCM) according to the equation of Tyrrell and Reid [20].

#### 2.2.2. Omasal and Total Tract Digestibility

During the last week of each period, cows entered the infusion and digestibility sampling phase. Cows were dosed twice daily, following milking, via the ruminal cannula with 10 g of Cr_3_O_2_, placed in cellulose bags (2 × 4 cm) starting 6 d before the digestibility trial until the end of each sampling period. Omasal sampling took place in one of the two Latin squares. The triple marker system was used to assess digesta flows at the omasal canal [21] using the following combination of markers: indigestible neutral detergent fiber (NDF) for the large particle solid phase (LP), YbCl_3_ for the small particle solid phase (SP), and Co-EDTA [22] for the fluid phase (FP). The YbCl_3_ was prepared by adding 230 g of Yb_2_O_3_ to 200 mL distilled water plus 320 mL concentrated HCl, heating and stirring until the solution was clear, and diluted up to 5 L with distilled water. The Co-EDTA and YbCl3 were infused into the rumen at the constant rate of 2.4 mL/min (providing 3.27 g of Co and 3.66 g of Yb daily) using an automated pump (model 33; Harvard Apparatus, Inc., Holliston, MA, USA). Each cow was given a priming dose of 4 L of the marker solution 60 h before sampling and a continuous infusion until the end of each sampling period.

During the digestibility trial, a sample of the TMR (300 g) was collected daily and frozen at −20 °C. Refusals of each animal were weighed, and a sample collected daily and frozen at −20 °C. Ruminal, omasal, and fecal samples were collected at 8 h intervals on 4 consecutive days of the digestibility trial with a 2 h shift between days. This sampling sequence provided 12 samples, one from every other hour of the 24 h day. In each sampling, a sample of 250 mL of rumen liquid and 50 mL of solid rumen content were taken from 4 different locations of the rumen (cranio-dorsal, cranio-ventral, ventral, caudoventral, and caudodorsal). Then, 100 mL of the rumen liquid were filtered through a double layer of cheese cloth, the pH was measured immediately in the filtrate (model 507, Crison Instruments SA, Alella, Spain), and the filtrate was pooled per cow and period and frozen at −20 °C. Then, the remaining 150 mL of rumen liquid were mixed with the 50 mL of solid rumen content, and bacteria were isolated using a combination of several detachment procedures [23] selected to obtain the maximum detachment without affecting cell integrity. Methylcellulose (15 mL of 20 g/L solution) was added to the mix, maintained in a waterbath at 38 °C for 30 min, and then placed in a fridge at 4 °C for 12 h. Samples were homogenized for 1 min at 24,000 rpm (Diax900, Heidolph, Nurnberg, Germany) to dislodge weakly attached bacteria and filtered through a double layer of cheese cloth. Solids that remained in the cheese cloth were washed with 50 mL of saline solution and the mixture was filtered again. The final filtrate was preserved by the addition of 7.15 mL of formaldehyde (0.37 *w/v*), pooled per cow and period, and refrigerated (4 °C) until the end of the period. When the last sample of the digestibility trial was added, solid and liquid associated bacterial cells were isolated, within 4 h, by differential centrifugation, first at 1000× *g* for 10 min to separate feed particles, and then the supernatant was centrifuged at 20,000× *g* for 20 min (Avanti J-20 XPI, Beckman Coutler, Indianapolis, IN, USA). Pellets were rinsed twice with saline solution and re-centrifuged at 20,000× *g* for 20 min. The final pellet was recovered with distilled water to prevent contamination of bacteria with ash. Bacterial cells were lyophilized and analyzed for DM, ash, nitrogen (N), and purine contents.

Omasal samples were obtained using the omasal sampling technique developed by Huhtanen, et al. [24] and Ahvenjärvi et al. [25], as adapted by Reynal and Broderick [26]. A tube passed through the ruminal cannula and was positioned in the omasal canal at each sampling according to the sampling schedule described above. Approximately 400 mL spot samples were collected, and 125 mL were pooled per cow and period and frozen at −20 °C. Fecal samples were collected at each sampling and 125 mL volume was pooled per cow and period and frozen at −20 °C. Urine was separated from feces by a device attached to the vulva of cows and directed with a plastic tube to a 25 L bottle containing 250 mL of sulfuric acid (0.1 *w/v*). When the bottle was filled, a second bottle replaced it. Every morning (24 h period), urine was weighed and an aliquot (0.1%) was sampled, composited per cow and period and frozen at −20 °C.

### 2.3. Sample Preparation

Samples of feeds, TMR, and TMR refusals were mixed per animal and period and divided in two portions. The first was placed in duplicate in an oven for 24 h at 103 °C for DM determination and the other portion was placed in an oven for 48 h at 55 °C and then ground with a cutting mill (SM 2000, Retsch GmbH, Haan, Germany) with a 1-mm screen for further analysis. Rumen and omasal samples were thawed at room temperature, homogenized for 1 min at 24,000 rpm (Diax900, Heidolph, Nurnberg, Germany), and subsamples were taken for ammonia-N and volatile fatty acid (VFA) analyses. Homogenized omasal samples were squeezed through 1 layer of cheesecloth, and the retained solids were defined as the omasal LP. The filtrate was centrifuged at 1000× *g* (5 °C, 5 min), and the supernatant was carefully separated from the pellet. The supernatant was defined as the omasal FP and the pellet was defined as the omasal SP. The separated phases were frozen, freeze dried, and ground through a 1-mm screen for analyses. Fecal pooled samples per cow and period were thawed at room temperature, homogenized for 2 min manually, dried in an oven for 48 h at 55 °C, and ground with a cutting mill (SM 2000, Retsch GmbH, Haan, Germany) with a 1-mm screen for further analyses.

### 2.4. Chemical Analyses

#### 2.4.1. Milk Composition

Fat, protein, lactose content, and somatic cell counts of milk samples were analyzed by near infrared spectroscopy (Associació Interprofessional Lletera de Catalunya; ALLIC, Cabrils, Spain). Frozen milk samples were thawed at room temperature and the milk FA profile was analyzed at ALLIC (Cabrils, Spain) with standardized methods: (i) ISO 14156 for fat extraction [27], (ii) ISO 15884 for fat esterification [28], and (iii) ISO 15885 for FA composition by gas-liquid chromatography [29]. Cows receiving CoEDTA infusions for the omasal sampling were excluded from this analysis because of the CoEDTA may affect milk FA synthesis [30,31].

#### 2.4.2. Marker Analyses

For the determination of omasal true digesta (OTD), concentrations of Co, Yb, and indigestible NDF were determined in LP and SP, and concentrations of Co and Yb in FP. The concentration of Co and Yb was analyzed using inductively coupled plasma optical emission spectroscopy on a Perkin-Elmer Optima 4300 DV instrument (Perkin-Elmer, Shelton, CT, USA). Samples of approximately 300 mg were added to 2.0 mL of distilled water plus 3.5 mL of a solution containing 0.69 *w/v* of HNO_3_ and diluted to a final volume of 50 mL with distilled water. Samples were digested in a high-pressure microwave reactor, reaching a final temperature/pressure of 250 °C/160 bar (MARSXpress; CEM Corporation, Matthews, NC, USA). For indigestible NDF analysis, samples of TMR offered and refused, LP, SP, and dried feces were incubated in duplicate in vitro for 240 h with rumen fluid from two dairy cows being fed a corn silage based TMR (on a DM basis: corn silage 446 g/kg, alfalfa silage 120 g/kg, corn meal 120 g/kg, soy plus 80 g/kg, soybean hulls 58 g/kg, wheat middlings 48 g/kg, dry citrus pulp dry 33 g/kg, chocolate meal 24 g/kg, energy booster 12 g/kg, dry molasses 9 g/kg, urea 21 g/kg, and a vitamin and mineral mix 29 g/kg). At the end of the fermentation, the contents of each flask were analyzed to determine the aNDFom content of the residue and filtered through crucibles (40-μm porosity) with the addition of microfiber glass filters as suggested by Raffrenato et al. [32] as adopted in omasal sampling experiments [33,34,35]. Residual aNDFom was analyzed according to Van Soest et al. [36] using a heat stable alpha-amylase and sodium sulfite, and expressed without residual ash. The freeze-dried FP, SP, and LP were mixed in the proper proportions for the reconstitution of the OTD flowing out of the rumen based on the triple marker method of France and Siddons [37].

Fecal dried samples were analyzed for Cr concentration according to Williams et al. [38]. Samples of OTD and bacterial cells were analyzed for purine content (adenine and guanine) by HPLC as described by Balcells et al. [39], using allopurinol as the internal standard.

#### 2.4.3. Component Analyses

Dry samples of TMR offered and refusals, OTD, duodenum, bacteria, and feces were ashed overnight at 550 °C in a muffle furnace [40], and OM was determined by difference. Samples of TMR, OTD, and feces were analyzed for aNDFom [36], using a heat stable alpha-amylase and sodium sulfite and expressed without residual ash. Total N of diets, OTD, bacteria, urine, and feces was determined by the Kjeldhal method [40]. Ammonia-N was analyzed by colorimetry as described by Chaney and Marbach [41], where 4 mL of a 0.2 N HCl solution were added to 4 mL of filtered rumen and omasal fluid and frozen. Samples were centrifuged at 3000 × *g* for 20 min, and the supernatant was used to determine ammonia-N by spectophotometry (Libra S21, Biochrom Technology, Cambridge, UK). Rumen samples for VFA analyses were prepared as described by Jouany [42] and analyzed by gas chromatography as described previously [16].

Fatty acids of diets and OTD were determined in 250 mg freeze dried sample as described by Vlaeminck et al. [43]. Fatty acid peaks were identified by comparing retention times with those of the corresponding standards [Supelco 37 Component FAME Mix, cis-11-Octadecenoic methyl ester, trans-11-Octadecenoic methyl ester (Supelco Analytical, Bellefonte, PA, USA); linoleic acid, conjugated methyl ester (Sigma-Aldrich, St. Louis, MO, USA)].

### 2.5. Calculation of Digestibility and Nitrogen Balance

Flows of DM into the omasum were calculated based on the triple marker method as described above, while fecal flow of DM was calculated using Cr as a single marker. Omasal flows were corrected for bacterial flow using purines as a microbial marker. Omasal true digestibility and total tract digestibility of nutrients (DM, OM, N, and aNDFom) were calculated as the difference between intake of nutrients and omasal and fecal flow of nutrients, respectively.

### 2.6. Statistical Analyses

All statistical analyses were conducted using JMP software of SAS (version 11; SAS Institute Inc., Cary, NC, USA). Data analysis was conducted with the following mixed effect models:

1. For analysis of rumen fermentation characteristics, total tract digestibility and N balance, the model accounted for the fixed effects of treatments, experimental periods and squares, their interactions, and the random effect of the cow (n = 6).
2. For omasal flows and rumen degradation, the same model was used without the square effect because the omasal sampling technique was performed with only one square (n = 3).
3. For analysis of milk yield and composition, model (1) was used. Moreover, the three sets of samples taken from each cow in each period were considered repeated measures and, therefore, the model included also day as a fixed effect (n = 6).
4. For the milk FA analysis, the square effect of model (1) was excluded due to the CoEDTA infusions performed for the omasal sampling (n = 3).

The significance of differences between means of treatments was declared at *p* ≤ 0.05, and trends at *p* ≤ 0.10 using the Tukey option.

## 3. Results

### 3.1. Animal Performance

Dairy cows consumed on average 20.7 kg of DM daily with a tendency towards higher intake for cows fed CAP and lower intake for those fed PTSO (*p* < 0.08; Table 2). Milk yield was not affected by treatment and ranged from 30.1 to 33.7 kg/d in CAP and PTSO, respectively. These differences resulted in higher feed efficiency in PTSO fed cows compared with CAP cows (1.65 vs. 1.41 kg of milk production/kg of DMI, respectively; *p* = 0.01). Milk protein concentration tended to be higher in CAP and lower in PTSO compared with CTR (34.0, 32.8, and 33.1 g/kg milk, respectively; *p* < 0.06). Lactose concentration was lower in PTSO compared with CTR (46.4 vs. 47.7 g/kg milk, respectively; *p* < 0.05). However, fat (average of 1.17 kg/d), protein (average of 1.04 kg/d), and lactose (average of 1.50 kg/d) yield were not affected by treatments. The FA profile in milk was not affected by treatments, with the exception of total polyunsaturated FA (PUFA), which was higher in PTSO fed cows (5.09 vs. 4.65 g/100 g of FA measured in PTSO and CTR, respectively; *p* < 0.04). Total saturated (SFA), monounsaturated, and unsaturated FA were not affected by treatments and averaged 69.5, 25.7, and 30.5 g/100 g of FA measured, respectively.

### 3.2. Ruminal Fermentation, and Omasal and Total Tract Digestibilities

Ruminal fermentation profile was not affected by treatments (Table 3). Average rumen pH (6.23), ammonia-N (11.8 mg/100mL), and total VFA concentration (129 mM) were similar among treatments. Acetate was the primary VFA (62.9 mol/100 mol of VFA), followed by propionate (20.8 mol/100 mol of VFA) and butyrate (12.7 mol/100 mol of VFA). Neither CAP nor PTSO affected the omasal flows of DM (13.0 kg/d), OM (10.6 kg/d), total N (423 g/d), ammonia N (12.3 g/d), NAN (411 g/d), microbial N (284 g/d), non-ammonia-non-microbial N (127 g/d), and aNDFom (3.8 kg/d; Table 4). Rumen degradability of true DM (409 g/kg), true OM (490 g/kg), aNDFom (362 g/kg), and CP (737 g/kg) was not affected by treatments. The efficiency of microbial protein synthesis was similar among treatments (34.0 g microbial N/kg fermented OM).

The addition of CAP decreased total tract apparent digestibilities of DM (684 and 659 g/kg for CTR and CAP, respectively; *p* < 0.03) and OM (728 and 705 k/kg for CTR and CAP, respectively; *p* < 0.01), mainly due to the reduction of aNDFom digestibility (518 and 454 g/kg for CTR and CAP, respectively; *p* < 0.04; Table 5). However, total tract CP apparent digestibility was not affected by treatments (average of 638 g/kg). Milk N use efficiency (g N in milk/g N intake) was not affected by treatments and ranged from 0.30 to 0.34 in CTR and PTSO, respectively.

## 4. Discussion

A large body of scientific evidence has been accumulated on the potential of EO as feed additives [3,4]. Given that the mode of action involves ruminal fermentation, most of these studies have been conducted in vitro. In vitro research provided useful information on the effects of EO on rumen microbial fermentation and to design in vivo experiments. However, in vivo studies have also revealed some effects associated with changes in DM intake and (or) postruminal effects [7,11,44]. This in vivo trial was designed to address digestive and cow’s performance effects in vivo.

### 4.1. Effects of Capsicum Oleoresin

The addition of CAP to in vitro batch fermentation resulted in changes in ammonia N concentrations and VFA profiles at low pH with high concentrate diets [5]. However, when tested in vivo in growing heifers fed high concentrate diets, the changes in the rumen fermentation profile were small and inconsistent [6,7,8]. The lack of effects of CAP on the rumen fermentation profile in the current trial agrees with results reported in dairy cattle supplemented with CAP at doses ranging from 250 to 1000 mg/d [9,10]. Some EO have modified ruminal biohydrogenation of fatty acids [45]. Therefore, we were also interested in exploring the potential effects of CAP on rumen biohydrogenation in vivo. However, omasal flows of individual and total SFA, UFA, monounsaturated FA, and PUFA in CAP supplemented cows were not different from CTR. We have no references where ruminal biohydrogenation has been measured in dairy cows supplemented with CAP. It is a general observation that many of the responses to EO observed in vitro are sometimes not replicated in vivo [9,10,46]. Differences in the effects of EO on rumen microbial fermentation in vitro vs. in vivo may be due to the low number or lack of protozoa, the fixed DMI relative to the fermentation volume, and the shorter adaptation time of in vitro studies; and issues related to the effective dose, the absorption of end products of fermentation through the rumen wall, and the dilution rate in in vivo studies, among others [47,48,49]. The addition of CAP had no effect on nutrient degradation in the rumen, but reduced total tract apparent DM and OM digestibility, mainly due to the reduced aNDFom total tract digestibility. Nutrient digestion in the rumen was determined only in one square, and lack of effect should be interpreted with caution because of low statistical power. However, the reduction in total tract apparent digestibility of aNDFom was measured in the two squares and it is likely the result of the trend for higher DMI. However, Oh et al. [44] reported a trend for lower DM and OM total tract digestibility without changes in DMI when capsicum was fed to dairy cattle protected from rumen fermentation. In contrast, Tager and Krause [9] and Oh et al. [10] reported no effect of CAP supplementation on apparent digestibility of DM and aNDFom. In the current study, we used Cr_3_O_2_ as a single external marker and spot sampling while others used undigestible NDF [10] or lanthanum [9], and spot sampling. To investigate if these contradicting results were due to methodological differences, we analyzed fecal DM flows using uNDF as described for the omasal samples. However, total flows of DM estimated with either uNDF or Cr3O2 were not different (on average 6.8 and 6.9 kg DM for uNDF and Cr_3_O_2_, respectively). Therefore, current results cannot be attributed to methodological differences of total tract digestibility markers. The identification of differences in total tract digestibilities compared with the lack of effects in nutrient degradation in the rumen may be attributed to the limited statistical power of the omasal sampling with only 3 cows.

Feeding CAP at doses ranging from 125 to 1000 mg/d to growing heifers resulted in consistent increases in DMI, ranging from 9% to 14% [6,7,8]. Therefore, we hypothesized that the addition of 500 mg/d of CAP may increase DMI in lactating dairy cattle. In this study, we detected a trend towards higher DMI in CAP fed cows compared with CTR. There are few experiments where CAP has been fed to dairy cows. Tager and Krause [9] supplemented lactating dairy cattle with 250 mg/d of capsicum and reported no effect on feed intake, but the dose used was lower than the one supplemented in this experiment (500 mg/d). Recently, CAP was tested in combination with monensin in lactating dairy cows and no effect on DMI was reported [50], but, again, doses were lower than those reported herein. However, even when CAP was fed at higher doses from 250 to 1000 mg/d, no effects on DMI were observed [10]. In all previously mentioned studies, CAP was fed unprotected from rumen microbial activity. Some research used a rumen protected form of CAP where post-ruminal effects could take place, but DMI was neither affected [11,44]. Therefore, it may be concluded that, in spite of the observed trend for increase in DM intake in the present trial, most evidence suggest that CAP does not affect DM intake in dairy cows.

Supplementation with CAP did not affect milk yield. Although Latin squares with long periods are not ideal experimental designs to investigate the effects on milk production because of the advancement of lactation, results agree with previous reports when CAP was supplemented unprotected from rumen activity at doses ranging from 250 to 1000 g/d [9,10]. In contrast, Oh et al. [11] observed a tendency for increasing milk yield when CAP was supplemented protected from rumen effects, and was attributed to an insulin mediated action. There were no effects of CAP on milk fat and lactose content, but milk protein content tended to increase compared with CTR. There is no clear hypothesis to justify this trend because neither protein degradation in the rumen, total tract CP digestibility, nor propionate proportions in the rumen were affected by CAP. Previous research reported no effects of feeding CAP on milk composition [9,10,11]. Neither the individual milk fatty acid profile (data not shown), nor the proportions of SFA, monounsaturated FA, PUFA, and UFA were affected by CAP. Oh et al. [10,50] reported no relevant effects on CAP on milk FA composition when fed alone or mixed with other phytonutrients. Overall, the supply of 500 mg/d of CAP to dairy cows had minimal effects on nutrient digestion and cattle performance.

### 4.2. Effects of Propyl-Propane Thiosulfonate

Garlic oil and their active components have been shown to modify the rumen fermentation profile in vitro [5,12,51]. The main active components of garlic oils are easily oxidized during exposure to air. The PTSO is a modified oxygen-stable organosulfurate compound derived from garlic that can be used in normal production processes without losing its activity. In previous studies, we tested increasing doses of PTSO on rumen microbial fermentation using a dual flow continuous culture fermenter system inoculated with rumen fluid from dairy cattle, and we reported changes in the fermentation profile that suggested its potential benefits as a modifier of ruminal fermentation [16,17]. However, there are no studies exploring the effects of PTSO on rumen microbial fermentation or production performance in dairy cows.

Results presented herein showed no effects of PTSO on total or individual VFA, nor in ammonia N concentrations, and agrees with Yang et al. [18] when garlic powder was fed to dairy cows. This is in contrast with previous dual flow continuous culture fermenters studies that reported that PTSO affected total VFA and their proportions [16,17]. Although nutrient flow to the omasum and the calculated degradation in the rumen were only measured in one square and may have limited statistical power, averages were very similar between PTSO and control. No effects on total tract digestibility of nutrients were observed. Supplying PTSO in vitro reduced ruminal biohydrogenation of FA, increasing the outflow of UFA at the expense of SFA [16,52,53]. Therefore, we hypothesized that the addition of PTSO may result in different omasal flows of FA and in altered milk FA profile. However, omasal flows of individual, total SFA, UFA, and monounsaturated FA in PTSO supplemented cows were not different than CTR. In general, there seems to be discrepancies between in vitro and in vivo studies, where the effects on microbial fermentation observed in vitro are often not replicated in vivo. The lack of effects in vivo vs. in vitro conditions may be attributed to several factors including the low number or lack of protozoa, the fixed DMI relative to the rumen volume, and the short adaptation time of in in vitro studies; and factor associated with the effective dose, the absorption of end-products of fermentation through the rumen wall, or the dilution rate in in vivo studies, among others [47,48,49].

In contrast to the lack of effects observed in CAP, the addition of PTSO tended to decrease DMI compared with CTR. Even though in vivo studies with PTSO are not available, few studies with lactating cows have tested other garlic compounds. Yang et al. [18] supplemented 5 g/d of garlic powder to lactating dairy cows and reported a similar DMI as in the current study (20.4 kg/d on average) with no difference among treatments. Blanch et al. [54] tested a mixture of cinnamaldehyde and garlic oil and reported no effect on DMI in the garlic supplemented dairy cows. Recently, Rossi, et al. [55] supplied dairy cows with diallyl-sulfide and garlic cloves and reported no difference in DMI among treatments. In addition, when garlic powder was fed to beef cattle no effect on DMI was reported [56,57]. However, a tendency to decrease DMI was reported when garlic was dosed post-ruminally [58], suggesting a possible mechanism of action not involving the rumen.

Feeding PTSO numerically increased milk yield by 2.2 kg/d, but differences were not significant compared with CTR. It should be observed that Latin squares with long periods required for digestibility studies are not the ideal experimental design to investigate the effects on milk production because of the inherent change in the stage of lactation. Previous studies with garlic or garlic oil compounds reported no significant effect on milk yield of dairy cows [18,54,55], although doses were, in all cases, lower. The combination of a trend for lower feed intake (*p* < 0.08) and the numerical increase in milk yield resulted in a 17.0% increase in feed efficiency (*p* < 0.01) in PTSO compared with CAP. Supplementation with PTSO did not affect milk fat and protein content. Although lactose concentrations decreased slightly, the biological significance of such a small change is not clear. These results generally agree with other trials where garlic products were fed to dairy cattle, with no effect on milk composition [18,58]. In contrast, the proportion of PUFA in PTSO were higher compared with CTR. These changes occurred in spite of lack of effect of PTSO on the omasal flow of PUFA. There are no studies where the effect of PTSO on the milk FA profile was investigated. However, in general, the few in vivo studies that have reported the relationship between EO and the milk or meat FA profile did not observe changes in the milk FA profile [10,46,59].

## 5. Conclusions

Neither CAP nor PTSO affected rumen fermentation, N metabolism, and N balance of dairy cows. Cows supplemented with CAP had reduced DM, OM, and aNDFom total tract digestibility probably due to the trend for increased DMI. However, we detected an increased milk efficiency (kg of milk/kg of DMI) in PTSO supplemented cows due to the numerically higher milk production and the trend for lower DMI.

## Figures and Tables

**Table 1:** Ingredients and chemical composition of the total mixed ration.

	g/kg DM	Fatty Acid	mg/kg DM
Ingredients		Saturated Fatty Acids	
Corn silage	318	C14:0	0.16
Dehydrated alfalfa	119	C15:0	0.04
Corn grain ground	196	C16:0	7.60
Soybean meal	173	C17:0	0.06
Soy hulls	117	C18:0	1.10
Beet molasses	32	C20:0	0.17
Megalac ^1^	12	C22:0	0.13
Vitamin and mineral mix ^2^	9	C23:0	0.07
NaCl	4.0	C24:0	0.15
MgO	2.5	Total	9.48
CaCO_3_	4.5	**Unsaturated Fatty Acids**	
NaHCO_3_	8.6	C15:1	0.10
Ca_2_PO_4_	4.4	cis-8 C16:1	0.12
**Nutrient composition**		cis-9 C16:1	0.01
Dry matter	529	C18:1n-9	6.35
Organic matter	916	cis-11 C:18:1	0.27
Crude protein	151	C18:2n-6	6.40
NDF	318	C18:3n-3	1.00
Metabolizable		Total	14.25
Energy ^3^, MJ/kg DM	2.61		

^1^ Calcium salts of palm oil fatty acids. ^2^ The vitamin and mineral mixture contained per kg of DM: 300 g of MgO; 267 g of urea; 33 g of S; 67 g of NaCl; 4660 mg of Zn; 2660 mg of Mn; 167 mg of Cu; 27 mg of Se; 33 mg of I; 7 mg of Co; 1000 kIU of vitamin A; 200 kIU of vitamin D3; and 1330 mg of vitamin E. ^3^ Calculated with the Cornell Net Carbohydrate and Protein System [19].

**Table 2:** Effect of propyl-propane thiosulfonate and capsicum oleoresin addition on dry matter intake, milk production, and milk composition of dairy cows (n = 6). ^1^

Item	CTR	CAP	PTSO	SEM	*p*-Value
DMI ^2^, kg/d	20.9	21.3	20.0	0.67	0.08
Milk yield, kg/d	31.5	30.1	33.7	3.10	0.11
FCM ^3^, kg/d	32.0	31.6	34.2	3.10	0.23
Milk true protein, kg/d	1.04	1.01	1.09	0.089	0.30
Milk fat, kg/d	1.14	1.16	1.21	0.129	0.36
Milk lactose, kg/d	1.50	1.43	1.57	0.159	0.24
Feed efficiency ^4^	1.51 ^ab^	1.41 ^b^	1.65 ^a^	0.138	0.01
Milk composition, g/kg					
Fat	36.3	38.8	36.8	1.89	0.11
True Protein	33.1	34.0	32.8	0.81	0.06
Lactose	47.7 ^a^	47.3 ^ab^	46.4 ^b^	0.30	0.05
SCC ^5^, 1000 cells/mL	485	154	397	186.4	0.18
Milk FA ^6^ profile, g/100 g FA measured					
SFA	71.48	70.97	65.96	2.020	0.23
MUFA	23.87	24.19	28.95	1.710	0.27
PUFA	4.65 ^b^	4.84 ^ab^	5.09 ^a^	0.528	0.04
UFA	28.52	29.03	34.04	2.020	0.23

^1^ CTR: control-no addition; CAP: capsicum oleoresin (500 mg/d); PTSO: propyl-propane thiosulfonate (250 mg/d); ^2^ DMI: dry matter intake. ^3^ FCM: 3.5% fat corrected milk production [20]. ^4^ Feed efficiency: kg of milk yield/kg of DMI. ^5^ SCC: somatic cell counts. ^6^ FA: fatty acid; SFA: saturated (C4:0, C6:0, C8:0, C10:0, C11:0, C12:0, C13:0, C14:0, C15:0, C16:0, C17:0, C18:0, C20:0, C21:0, C22:0, C23:0, C24:0) FA; MUFA: Monounsaturated FA (C12:1, C14:1, C16:1, C17:1, trans-9 C18:1, trans-10 C18:1, trans-11 C18:1, C18:1 n-9, C20:1, C22:1, C24:1); PUFA: Polyunsaturated FA (cis-9 trans-11 CLA, C18:2n-6, C20:2, C18:3n-6, C18:3n-3, C20:3n-6, C20:4n-6, C20:5n-3, C22:5n-3); UFA: unsaturated FA (MUFA, PUFA). This analysis conducted in 3 out of 6 cows. ^a,b^ Means in the same row with different superscript differ (*p* < 0. 05).

**Table 3:** Effect of propyl-propane thiosulfonate and capsicum oleoresin addition on rumen fermentation characteristics of lactating dairy cow (n = 6) ^1^.

Item	CTR	CAP	PTSO	SEM	*p*-Value
Average pH	6.2	6.2	6.3	0.09	0.57
NH_3_-N, mg/100 mL	12.7	11.7	11.2	1.23	0.57
VFA ^2^, mM	132	129	126	5.5	0.77
VFA profile, mol/100 mol					
Acetate	63.2	62.7	62.7	0.88	0.93
Propionate	20.4	20.9	21.0	0.67	0.83
Butyrate	12.7	12.9	11.9	0.51	0.43
Valerate	1.5	1.6	1.7	0.15	0.44
BCVFA ^3^	1.5	1.3	1.6	0.19	0.62

^1^ CTR: control-no addition; CAP: capsicum oleoresin (500 mg/d); PTSO: propyl-propane thiosulfonate (250 mg/d); ^2^ VFA: volatile fatty acids. ^3^ BCVFA: branched chained VFA.

**Table 4:** Effect of propyl-propane thiosulfonate and capsicum oleoresin addition on intake, flows at the omasal canal, and ruminal degradability of dairy cows (n = 3) ^1^.

Item	CTR	CAP	PTSO	SEM	*p*-Value
Dry matter					
Intake, kg/d	18.7	18.9	18.0	0.9	0.29
Omasal flow, kg/d	12.7	13.3	12.9	0.36	0.24
True degradability, g/kg	415	408	404	13	0.78
Organic matter					
Intake, kg/d	17.4	17.4	16.6	0.8	0.31
Omasal flow, kg/d	10.5	10.9	10.5	0.3	0.44
True degradability, g/kg	489	489	492	11	0.97
Nitrogen (N)					
Intake, g/d	488	492	464	23	0.23
Omasal flow, g/d	415	427	427	17	0.86
Ammonia N flow, g/d	12.4	13.0	11.4	0.9	0.62
Non-ammonia N flow, g/d	403	415	415	17	0.85
Microbial N flow, g/d	267	299	285	16	0.50
NANM ^2^ N flow, g/d	136	115	130	18	0.60
True degradability, g/kg	721	763	727	37	0.70
EMPS ^2^, g N/kg OMTD	31.5	35.5	35.2	3.5	0.66
aNDFom					
Intake, kg/d	6.0	6.2	5.9	0.3	0.29
Omasal flow, kg/d	3.7	4.0	3.7	0.2	0.52
Degradability, g/kg	374	344	369	24.9	0.66
Fatty acids ^3^					
Intake, g/d	444	449	428	23	0.53
SFA omasal flows, g/d	393	371	351	22	0.48
UFA omasal flows, g/d	119	125	115	8	0.52
Total FA omasal flows, g/d	513	496	465	24	0.47

^1^ CTR: control-no addition; CAP: capsicum oleoresin (500 mg/d); PTSO: propyl-propane thiosulfonate (250 mg/d); ^2^ NANM: Non ammonia non microbial nitrogen; Efficiency of microbial protein synthesis: g of microbial N/kg true fermentable organic matter. ^3^ FA: Fatty acids, SFA: Saturated FA, UFA: Unsaturated FA.

**Table 5:** Effect of propyl-propane thiosulfonate and capsicum oleoresin addition on the apparent total tract digestibility (g/kg) of dairy cows (n = 6) ^1^.

Item	CTR	CAP	PTSO	SEM	*p*-Value
Dry matter	684 ^a^	659 ^b^	682 ^a^	8.4	0.03
Organic matter	728 ^a^	705 ^b^	726 ^a^	7.0	<0.01
Crude protein	649	626	640	10.6	0.20
aNDFom	518 ^a^	454 ^b^	525 ^a^	17.8	0.04

^1^ CTR: control-no addition; CAP: capsicum oleoresin (500 mg/d); PTSO: propyl-propane thiosulfonate (250 mg/d); ^a,b^ Means in the same row with different superscript differ (*p* < 0. 05).
